# Association between polymorphisms in *RMI1*, *TOP3A*, and *BLM *and risk of cancer, a case-control study

**DOI:** 10.1186/1471-2407-9-140

**Published:** 2009-05-11

**Authors:** Karin Broberg, Elizabeth Huynh, Karin Schläwicke Engström, Jonas Björk, Maria Albin, Christian Ingvar, Håkan Olsson, Mattias Höglund

**Affiliations:** 1 Department of Occupational and Environmental Medicine, Lund University, Lund, Sweden; 2 Competence Centre for Clinical Research, Lund University Hospital, Lund, Sweden; 3 Department of Surgery, Lund University, Lund, Sweden; 4 Department of Oncology, Lund University, Lund, Sweden; 5 Department of Clinical Genetics/SCIBLU Genomics, DNA Microarray Resource Centre Lund University, Lund, Sweden

## Abstract

**Background:**

Mutations altering BLM function are associated with highly elevated cancer susceptibility (Bloom syndrome). Thus, genetic variants of BLM and proteins that form complexes with BLM, such as TOP3A and RMI1, might affect cancer risk as well.

**Methods:**

In this study we have studied 26 tagged single nucleotide polymorphisms (tagSNPs) in *RMI1*, *TOP3A*, and *BLM *and their associations with cancer risk in acute myeloid leukemia/myelodysplatic syndromes (AML/MDS; N = 152), malignant melanoma (N = 170), and bladder cancer (N = 61). Two population-based control groups were used (N = 119 and N = 156).

**Results:**

Based on consistency in effect estimates for the three cancer forms and similar allelic frequencies of the variant alleles in the control groups, two SNPs in *TOP3A *(rs1563634 and rs12945597) and two SNPs in *BLM *(rs401549 and rs2532105) were selected for analysis in breast cancer cases (N = 200) and a control group recruited from spouses of cancer patients (N = 131). The rs12945597 in *TOP3A *and rs2532105 in *BLM *showed increased risk for breast cancer. We then combined all cases (N = 584) and controls (N = 406) respectively and found significantly increased risk for variant carriers of rs1563634 A/G (AG carriers OR = 1.7 [95%CI 1.1–2.6], AA carriers OR = 1.8 [1.2–2.8]), rs12945597 G/A (GA carriers OR = 1.5 [1.1–1.9], AA carriers OR = 1.6 [1.0–2.5]), and rs2532105 C/T (CT+TT carriers OR = 1.8 [1.4–2.5]). Gene-gene interaction analysis suggested an additive effect of carrying more than one risk allele. For the variants of *TOP3A*, the risk increment was more pronounced for older carriers.

**Conclusion:**

These results further support a role of low-penetrance genes involved in BLM-associated homologous recombination for cancer risk.

## Background

Bloom syndrome is a condition characterised by growth inhibition, light sensitivity, and high incidence of cancer in early life [1]. Although there appears to be a predominance of lymphocytic leukemia and lymphoma, many cancer types are seen in this condition. A defining feature of Bloom's syndrome is an elevated frequency of sister chromatide exchanges. These arise from crossing over of chromatide arms during homologous recombination, a ubiquitous process that exists to repair DNA double-stranded breaks and damaged replication forks. Whereas crossing over is required in meiosis, it can in mitotic cells be associated with a detrimental loss of heterozygosity, a common feature in neoplastic cells.

BLM, the helicase mutated in Bloom syndrome, is found in protein complexes together with topoisomerase IIIa (TOP3A) and a newly identified member, the RECQ-mediated genome instabilitity 1 (RMI1) protein, that process double Holliday junction intermediates into non-crossover recombinants [2-5]. This dissolution activity of the BLM-TOP3A-RMI1 complex is thought to be critical for the suppression of DNA crossover formation in mitotic cells and cancer avoidance in humans. The complex might process many other DNA structures as well, such as stalled replication forks [6] and has been implicated in checkpoint signalling and checkpoint responses during DNA damage [5].

Since mutations that alter BLM function are associated with elevated cancer susceptibility, we reasoned that genetic variants of *BLM *and other proteins that form a complex with BLM might affect the risk for different cancer forms. In order to test this hypothesis we analysed in this study polymorphisms in the *RMI1*, *TOP3A *and *BLM*, and their association with cancer risk in available case-control materials, namely AML/MDSs (acute myeloid leukemia and myelodysplastic syndromes), malignant melanoma, and bladder and breast cancer.

## Methods

### Study populations

The studies have been approved by the Ethics Committee of Lund University. All study participants gave informed consent before participation in the study. We did not ask for ethnicity of the study participants. However, based on their names, the absolute majority of the participants was of European descent (predominating Swedish). All cancer patients were voluntarily recruited from the Southern Health Care Region of Sweden. Study population characteristics for different cancer forms are shown in Table 1. The AML/MDS and the malignant melanoma study populations are described in more detail in Broberg et al. [7]. In short, the diagnoses were: 78 cases of AMLand, 56 cases of MDS, and in 18 subjects AML preceeded by a MDS. All the participating melanoma patients (N = 170) had a primary cutaneous melanoma diagnosed except for two patients, who presented with metastatic disease without known primary site of their melanoma. The AML/MDS patients' samples were collected during the years 1998–2004 and the malignant melanoma samples during 2001–2005. The control group was drawn randomly from the Regional Population Registry during 2001–2004, frequency matched with the cases series with respect to sex, year of birth, as well as country of living. The bladder study groups have been studied earlier and are described more in detail in Broberg et al. [8]. The bladder cancer patients' (N = 61) and the control individuals' samples (N = 156) (mouth washes) were collected during 1995–2000. The control group was drawn randomly from the Regional Population Registry during 1995–2000, frequency matched with the cases with respect to sex, year of birth, as well as country of living. The breast cancer patients all came for treatment at the Oncology Clinic at Lund University Hospital between 1990 and 1998, where they accepted to participate in the study and donated blood samples. The case group encompassed 200 patients, all of which were women. According to the TNM classification, 6 individuals were in stage 0, 79 in stage I, 72 in stage II and 15 in stage III. Twenty-eight cases could not be classified, mainly because of no axillary sampling performed due to locally advanced tumors. The control group consisted of 131 accompanying female spouses to cancer patients coming to the Oncology Clinic for treatment, recruited from Southern Sweden. The control individuals filled in a questionnaire about their health status and donated a blood sample. Spouses without former cancer were selected for the study.

**Table 1:** Study characteristics of cases and controls for the different cancer forms.

	AML/MDS^a^	Malignant melanoma		Bladder cancer		Breast cancer	
	Cases	Cases	Controls	Cases	Controls	Cases	Controls

N	152	170	119	61	156	200	131
Median age (range)	72 (22–95)	66 (24–80)	68 (22–96)	69 (35–85)	69 (21–90)	59 (34–86)	63 (23–85)
Women/Men (%)	73/79 (48/52)	87/83 (51/49)	53/66 (44.5/55.5)	9/52 (15/85)	34/122 (22/78)	200/0 (100/0)	131/0 (100/0)

^a ^AML/MDS denotes acute myeloid leukemia/myelodysplastic syndromes

### Polymorphism selection and initial screening

Polymorphisms were selected in Bloom syndrome related genes (*RMI1*, *TOP3A *and *BLM*) from the HapMap data http://www.hapmap.org. TagSNPs were identified from the CEU population data (CEPH, Utah residents with ancestry from northern and western Europe), and by running the data in Haploview ([9]). The TagSNPs chosen showed an allelic frequency of at least 10%. DNA extractions for all samples were made with QIAmp 96 DNA blood kit (Qiagen, Hilden, Germany) by SWEGENE resource centre for Profiling Polygenic Diseases in Malmö University hospital, Sweden, apart from the blood samples from the breast control group, which were extracted for DNA with Wizard Genomic DNA Purification Kit (Promega, Madison, WI, USA). The polymorphisms were analyzed with matrix-assisted laser desorption/ionization time-of-flight

(MALDI-TOF) mass spectrometry (Sequenom™, San Diego, CA) at the SWEGENE resource centre for Profiling Polygenic Diseases in Malmö University hospital. For the Sequenom assays, 20 percent of the samples were rerun and negative controls (water instead of DNA) were included in each run. Out of 28 tagSNPs analysed, 2 failed in the analysis, which left us with 26 tagSNPs. The frequency of individuals with missing genotype data ranged between 1.3%–6.8%/SNP assay for leukaemia and malignant melanoma, and 7.7%–12.7%/SNP assay for bladder cancer. The drop rate for bladder cancer probably reflects that there was limited amount of DNA left. We carefully evaluated the results of each assay and only included those samples that demonstrated positive signals in most assays. Selection of tagSNPs for further analysis of the breast cancer study population was based on changes in effect estimates (computed as odds ratios, ORs) in the same direction (reduced or increased risk) in at least two cancer forms of the three analyzed. Secondly, the allele frequencies in the two control groups should be similar.

### Genotyping of polymorphisms in the breast cancer/control material

The genotyping was performed on 200 cases of breast cancer and 131 controls. The selected polymorphisms were rs1563634 and rs12945597 (C__31923586_10) in *TOP3A*, rs401549 and rs2532105 in *BLM*. Taqman assays and allelic discrimination was run on an ABI PRISM 7000 (Applied Biosystems). The Taqman assay reaction volume was 25 μl containing: 1× Universal Taqman mix (Applied Biosystems), each primer at 0.45 μM mixed with each probe at 0.10 μM, and 5–12 ng of DNA template. The thermal cycle protocol was 95°C for 10 min, 95°C for 15 sec and 60°C for 1 min (40 cycles). Plate reading for allelic discrimination was performed under 60°C for 1 min. For all assays, at least 5% of the samples were reanalyzed and the concordance rate of these analyses was 100%.

### Statistical analysis

The Hardy-Weinberg equilibrium (HWE) test was undertaken using the chi-square test in the control groups. Effect estimates were computed as odds ratios (ORs) with 95% confidence intervals (95% CIs) by logistic regression using SPSS 14.0 (version 14; SPSS, Chicago, IL, USA). For rs1563634 in *TOP3A*, the reference category was changed in Tables 2, 3, 4 and 5 to the variant homozygous genotype in order to display cancer risk increment. The analyses were performed both without any adjustments, as well as with adjustments for sex and age. When stratifying for age, subjects were categorized into two groups, based on the median age among the cancer cases and control groups combined as a cut-off (< 65 and ≥ 65 years). The stratified analysis was adjusted for sex.

**Table 2:** Associations of polymorphisms in A) *TOP3A *and B) *BLM *and cancer risk (presented as odds ratios and 95% confidence interval) in different cancer forms.^a^

Cancer type	Polymorphism	Risk allele % Cases/controls	Genotype	Cases	Controls	OR	95% CI
AML/MDS	***TOP3A*** **rs1563634^b^**	75/66	AA	11	14	1.0^c, d^	-
			AG	54	52	1.3	0.53–3.1
			GG	84	52	2.0	0.82–4.7
Malignant melanoma		75/66	AA	12	14	1.0	-
			AG	60	52	1.4	0.58–3.2
			GG	96	52	2.2	0.93–5.1
Bladder		68/67	AA	3	19	1.0	-
cancer			AG	32	54	**3.9**	**1.1–14**
			GG	25	66	2.5	0.67–9.1
Breast cancer		68/68	AA	19	16	1.0	-
			AG	88	48	1.5	0.72–3.2
			GG	92	66	1.2	0.57–2.5
AML/MDS	***TOP3A*** **rs12945597**	37/25	GG	61	64	1.0	-
			GA	63	49	1.3	0.76–2.2
			AA	22	5	**4.9**	**1.7–14**
Malignant melanoma		32/25	GG	78	64	1.0	-
			GA	72	49	1.2	0.74–2.0
			AA	18	5	**3.0**	**1.0–8.4**
Bladder		36/30	GG	26	73	1.0	-
cancer			GA	25	49	1.4	0.75–2.8
			AA	9	17	1.5	0.58–3.7
Breast cancer		31/26	GG	96	76	1.0	-
			GA	84	40	**1.7**	**1.0–2.7**
			AA	20	15	1.1	0.52–2.3
AML/MDS	***BLM*** **rs401549**	31/30	AA	65	57	1.0	-
			AG	75	51	1.3	0.77–2.1
			GG	9	10	0.77	0.29–2.0
Malignant melanoma		36/30	AA	62	57	1.0	-
			AG	90	51	1.6	0.98–2.7
			GG	16	10	1.5	0.61–3.5
Bladder		37/30	AA	26	64	1.0	-
cancer			AG	24	67	0.90	0.47–1.7
			GG	10	8	**3.1**	**1.1–8.7**
Breast cancer		34/33	AA	87	58	1.0	-
			AG	94	61	1.0	0.65–1.6
			GG	19	12	1.1	0.48–2.4
AML/MDS	***BLM*** **rs2532105**	16/12	CC	103	90	1.0	-
			CT+TT^e^	46	27	1.4	0.83–2.5
Malignant melanoma		18/12	CC	109	90	1.0	-
			CT+TT	59	27	**1.8**	**1.1–3.2**
Bladder cancer		20/10	CC	40	111	1.0	-
			CT+TT	20	28	**2.1**	**1.0–4.2**
Breast cancer		17/13	CC	133	104	1.0	-
			CT+TT	67	27	**2.0**	**1.2–3.3**

^a ^Observe that the control group for AML/MDS (acute myeloid leukemia/myelodsyplastic syndromes) and malignant melanoma case groups was the same. Logistic regression, adjusted for age and sex. Statistically significant associations (p ≤ 0.05) are denoted in bold.

^b^Accession number for polymorphisms in the SNP database of National Centre of Biotechnology Information, http://www.ncbi.nlm.nih.gov/sites/entrez?db=snp

^c ^Reference category.

^d ^For rs1563634, the reference category was changed to the variant allele in order to display cancer risk increment, and the risk allele frequency denotes thus the more common allele.

^e ^Due to low variant allelic frequencies, the variant genotypes were merged.

**Table 3:** Cancer risk estimates for gene-gene combinations of A) rs12945597 (*TOP3A*) and rs2532105 (*BLM*) and B) rs1563634 (*TOP3A*) and rs2532105 (*BLM*) in different cancer forms.^a^

IIIA. rs12945597+rs2532105			
	Genotype	OR	95% CI
AML/MDS	GG+CC	1.0	-
	GG+T-	1.4	0.61–3.0
	A-+CC	1.6	0.89–2.8
	A-+T-	**2.4**	**1.1–5.4**
Malignant melanoma	GG+CC	1.0	-
	A-+CC	1.7	0.98–3.0
	GG+T-	**2.5**	**1.2–5.2**
	A-+T-	**2.4**	**1.1–5.4**
Bladder cancer	GG+CC	1.0	-
	A-+CC	1.4	0.68–2.9
	GG+T-	2.1	0.74–6.1
	A-+T-	**3.0**	**1.1–7.7**
Breast cancer	GG+CC	1.0	-
	A-+CC	1.5	0.92–2.6
	GG+T-	**2.0**	**1.0–4.1**
	A-+T-	**3.0**	**1.4–6.4**

IIIB. rs1563634^b^+rs2532105			

	Genotype	OR	95% CI

AML/MDS	AA+CC	1.0	-
	AA+T-	0.48	0.087–2.7
	G-+CC	1.1	0.39–3.1
	G-+T-	1.9	0.62–5.8
Malignant melanoma	AA+CC	1.0	-
	AA+T-	0.48	0.089–2.6
	G-+CC	1.1	0.41–3.1
	G-+T-	2.5	0.84–7.4
Bladder cancer	AA+CC	1.0	-
	AA+T-	1.7	0.12–23
	G-+CC	3.0	0.64–14
	G-+T-	**6.5**	**1.3–33**
Breast cancer	AA+CC	1.0	-
	G-+CC	**2.8**	**1.1–7.2**
	AA+T-	**12**	**2.1–70**
	G-+T-	**4.6**	**1.7–13**

^a ^Statistically significant associations (p ≤ 0.05) are denoted in bold. The analyses were adjusted for age and sex.

^b ^For rs1563634, the reference category was changed to the variant allele in order to display cancer risk increment.

**Table 4:** Association of polymorphisms in *TOP3A *and *BLM *and risk of cancer (all cancer cases and all controls combined).^a^

SNP	Genotype	OR	95% CI	FPRP 0.1^b^	FPRP 0.01
rs1563634^c^	AA	1.0	-	-	
(*TOP3A*)	**AG**	**1.7**	**1.1–2.6**	**0.48**	**0.92**
	**GG**	**1.8**	**1.1–2.8**	**0.29**	**0.84**
rs12945597	GG	1.0	-	-	-
(*TOP3A*)	**AG**	**1.5**	**1.1–1.9**	**0.014**	**0.13**
	**AA**	**1.6**	**1.0–2.5**	**0.36**	**0.89**
rs401549	AA	1.0	-	-	-
(*BLM*)	AG	1.2	0.89–1.5	-	-
	GG	1.3	0.82–2.2	-	-
rs2532105	CC	1.0	-	-	-
(*BLM*)	**CT+TT**	**1.8**	**1.5–2.5**	**0.029**	**0.27**

^a ^Statistically significant associations (p ≤ 0.05) are denoted in bold. The analyses were adjusted for age and sex.

^b ^FPRP is only calculated for significant p-values.

^c ^For rs1563634, the reference category was changed to the variant allele in order to display cancer risk increment.

**Table 5:** Gene-gene associations of polymorphisms in *TOP3A *and *BLM *and risk of cancer (all cancer cases and all controls combined).^a^

Polymorphisms	Genotype	OR	95% CI
rs12945597+rs2532105	GG+CC	1.0	-
	A-+CC	**1.6**	**1.2–2.1**
	GG+T-	**2.0**	**1.3–3.1**
	A-+T-	**2.7**	**1.8–4.3**
rs1563634+rs2532105	AA+CC	1.0	-
	G-+CC	**1.9**	**1.1–3.2**
	AA+T-	2.0	0.85–4.
	G-+T-	**3.5**	**2.0–6.3**

^a ^Statistically significant associations (p ≤ 0.05) are denoted in bold. The analyses were adjusted for age and sex.

To assess false positives, the False Positive Report Probability (FPRP) [10] was calculated, based on observed association data, according to the formula; α *(1-π)/(α*(1-π) + (1-β)*π), α denotes the p-values from the logistic regression analyses, 1-β denotes the statistical power for the tests and π denotes the prior probability of a true association of the tested genetic variant and outcome. OR values above 1.5 were considered as this is a likely threshold value for important biologic effects [11,12]. The prior probability employed was set to 0.01 (low probability) and 0.1 (high probability) for all SNPs. The FPRP treshold was set to 0.5, thus statistically significant SNPs with a FPRP above 0.5 were in this study not considered reliable to classify as true positives. These values, as well as the values for ORs and 95% CIs were entered into the online spreadsheet included in Wacholder et al. [10] for FPRP calculations.

### Bioinformatics

SNPs with a statistically significant association were further evaluated *in silico *for potential function. PupaSNP (http://www.pupasnp.org, [13,14]) was employed in order to detect SNPs potentially affecting transcription factor binding sites, exonic splicing enhancers/silencers, triplexes, splice sites and microRNA target sites. Emboss CpGPlot http://www.ebi.ac.uk/emboss/cpgplot/ was employed to detect CpG-rich areas.

## Results

All SNPs were in HWE in the control groups for AML/MDS, malignant melanoma, and bladder cancer. The four selected SNPs were in HWE in the breast cancer control group as well (data not shown). The results from the initial screening of all SNPs and their association with AML/MDS, malignant melanoma, and bladder cancer, are shown in Additional file 1. Few statistically significant findings were obtained when the tumour types were analysed separately. The SNP rs296887 (*RMI1*) was significantly associated with increased cancer risks for AML/MDS, as well as for malignant melanoma, but not with risk for bladder cancer. In *TOP3A*, rs12945597 was associated with increased risk for AML/MDS and malignant melanoma, and showed a non-significantly increased risk for bladder cancer. In *BLM*, rs401549 was associated with significantly increased risk for bladder cancer and a non-significantly increased risk for malignant melanoma. Rs2532105 (*BLM*) showed increased risk for malignant melanoma and bladder cancer, and a non-significant risk increment for AML/MDS. In AML/MDS, rs393974 (*BLM*) and rs6496724 (*BLM*) were also significantly associated with cancer risk.

For further analysis of breast cancer, rs12945597 in *TOP3A*, rs401549, and rs2532105 in *BLM *were selected, as well as rs1563634 in *TOP3A*, which showed similar non-significant protective effects in all three cancer forms for the variant homozygous carriers. For rs1563634, the reference category was in the subsequent analyses changed to the variant homozygotes in order to display cancer risk increment. The rs12945597 in *TOP3A *and rs2532105 in *BLM *(Table 2) was significantly associated with increased breast cancer risk. Dual-polymorphisms analyses (in two different genes) were performed for all cancer forms for the three SNPs showing main genetic effects (Table 3). An allele-dosage effect was found for the combination rs12945597 (*TOP3A*) and rs2532105 (*BLM*) for AML/MDS, bladder and breast cancer, where carriers with two variant alleles displayed the highest risks (Table 3A).

Based on these results, we combined all cases (N = 584) and controls (N = 406), respectively and performed association analyses. The cases and controls showed a similar distribution in age (cases median age = 65 years, controls = 64 years), but differed for sex (63% women among cases and 54% women among controls). For rs1563634, rs12945597, and rs2532105, a significantly increased risk for developing cancer was found (Table 4). The highest risk was found for the combination of rs1563634 in *TOP3A *and rs2532105 in *BLM *(OR = 3.5, 95% CI 2.0–6.3; Table 5). We also performed a three-polymorphism combination analysis for *TOP3A *rs1563634 and rs12945597, and *BLM *rs2532105 and found an OR = 3.8, 95% CI 2.0–7.3, for carriers of three variant alleles.

The impact of age on the associations between genetic markers of the BLM-complex and cancer risk was evaluated. For the two polymorphisms in *TOP3A *a stronger effect was found among elderly individuals (rs1563634 GG carriers ≥ 65 years OR = 2.2 95% CI 1.2–4.0, <65 years OR = 1.4 95% CI 0.73–2.7; rs12945597 AA carriers ≥ 65 years OR = 2.3 95% CI 1.2–4.4, <65 years OR = 1.1 95%CI 0.58–2.0). No such effect could be detected for the two variants analysed in *BLM *(data not shown).

We evaluated the significance of the findings for all cancer cases and controls combined by using FPRP analysis (Table 4). At high prior probability (set as 0.1) all statistically significant findings demonstrated a low probability for false positive results. However, at a lower prior probability (0.01), only the *TOP3A *rs12945597 (heterozygote) and *BLM *rs2532105 displayed a FPRP value < 0.5.

The bioinformatics analyses revealed that *TOP3A *rs1563634 is situated in a CpG island about 700 base pairs upstream to the start codon in *TOP3A. TOP3A *rs12945597 is located approximately 5 Kbp downstream the coding region. *BLM *rs401549 and rs2532105 are situated in intron 21 of the gene.

## Discussion

The present study shows that individuals carrying genetic variants of the BLM-TOP3A-RMI1 complex have an increased risk of AML/MDS, malignant melanoma, bladder and breast cancer. The strongest genetic risk marker was found in *BLM*, i.e., the variant allele of rs2532105, which showed a statistically significantly increased risk for three out of four cancer forms analyzed. By considering two polymorphisms a stronger association was obtained, but there were no indications of a multiplicative interaction between genetic variants at different gene loci.

These data suggests that some polymorphisms in the BLM complex are general cancer susceptibility markers and that the homologous recombination system may be involved in neoplastic transformation of several cell types. Our finding is in line with the observation that individuals with Blooms syndrome carrying mutations in *BLM*, essential for the homologous recombination complex, show elevated risk for various cancer types. There are increasing amount of evidence that homologous recombination generates loss of heterozygosity in various cancer types e.g. acute myeloid leukemias, follicular lymphomas, breast cancer, bladder cancer, gastrointestinal stromal tumors and Barrett's oesophagus [15-20]. A recent study have also shown the importance of the BLM-TOP3A-RMI1 for the maintenance of the genome stability by faithful chromosome segregation and prevention of anaphase bridges[21], a cytogenetic aberration found in several types of neoplastic cells. In a recent investigation, we found a more pronounced effect among elderly individuals for the Ser455Asn polymorphism in *RMI1 *[7]. We found a similar pattern in this study for the two polymorphisms in *TOP3A*, possibly reflecting the finding that mitotic recombination increases with age [22] and, hypothetically, that protein variants involved in this process has a larger influence when the body burden of mitotic recombination increases.

The effect of the polymorphisms remains to be clarified. None of the three rs1563634, rs12945597, and rs2532105 result in non-synonymous exchanges or are in LD with non-synonymous SNPs (data not shown). However, rs1563634 is positioned in a CpG island upstream to the *TOP3A *gene. The SNP does not in itself introduce or remove an extra CpG, but CpG regions tend to be associated with gene promoter regions and hence the polymorphism may affect transcriptional activity [23].

There are several limitations with the study. One problem is that smaller case-control studies may lead to spurious association and it is important to stress that our findings need to be confirmed in other case-control studies. For bladder cancer, the drop out in genotype data/assay was fairly high and these data should thus be cautiously interpreted. The characteristics of the control groups differed; they were selected in different ways during different time frames, showed varying degree of participation, and varied in sex and age distribution. However, allelic frequencies in the different control groups were similar, indicating that the observed frequencies are good estimates of the true frequency in this region of Sweden. On the other hand, there were different allelic distributions for the SNPs in the different cancer forms. Different repair pathways are important for different cancer forms, but based on our results on each cancer form, homologous recombination may be important in several cancer types. However, the genetic influence is not expected to be exactly the same for the different cancers and therefore, the allelic frequencies may differ. Since the separate analysis for each cancer points at influence of the BLM system, it was considered acceptable to combine all cases and controls, despite the fact that the cancer forms in this study are very different in etiology, and genetic heterogeneity is present in different tumour forms. When the cases from all cancer forms and the different control groups were combined in one analysis, the case and control groups differed somewhat in sex, but were very similar in age distribution. Still, it should be noted that apart from factors as age and sex, we have not evaluated cancer-specific risk factors, which could influence the associations between SNPs in the BLM complex and cancer risk.

We conducted a false-positive report probability (FPRP) analyses in order to assess the risk of false positives. The prior probabilities were considered relatively high (0.1) due to the impact of mutations in *BLM *for cancer risk in Bloom syndrome patients and the functional relevance of the TOP3A for the BLM complex. However, since there was little evidence for functional effect for the SNPs analysed, based on bioinformatics analysis, the prior probability of 0.01 was also taken into consideration. With this cut-off, only the *TOP3A *rs12945597 and *BLM *rs2532105 remained as significant findings. Thus, the other observed associations in this study have to be validated in a larger cohort.

There are very few other studies of polymorphisms of the BLM-complex and cancer risk. As mentioned, we have previously studied the Ser455Asn polymorphism in *RMI1 *and found an association between increased risk of AML/MDS and malignant melanoma for variant carriers. In this study, we analysed the SNP rs296887, which is linked to *RMI1 *Ser455Asn and found a similar but weaker effect for AML/MDS and malignant melanoma but no effect in bladder cancer. Other studies have analyzed *BLM *Thr298Met and small cell lung carcinoma risk [24] and *BLM *Pro868Leu and familial breast cancer [25], but none of these polymorphisms showed any association with cancer risk. However, there are several studies on other genes that are required for homologous recombination repair, i.e. *RAD51*, *XRCC2 *and *XRCC3*. The *RAD51*-135 (G/C) variant allele has been associated with increased risk of AML and this relation was most pronounced for AML associated with a previous history of treatment with chemo- or radiotherapy [26-29]. The results for the *XRCC3 *Thr241Met variant have so far been not been conclusive: variant carriers was found to have increased risk of bladder and breast cancer and malignant melanoma [30-32], whereas in a previous study of ours no association with this marker and bladder cancer was demonstrated [8]. In one meta-analysis, there was no significant associations between *XRCC3 *Thr241Met and cancer of the bladder, breast, lung or skin [33], but in another meta-analysis the Met/Met genotype showed a small cancer risk with the strongest effect found for breast cancer [34]. Also for the *XRCC2 *gene the results have been contradictory, the variant allele of Arg188His has been associated with increased cancer risk for breast and pharyngeal cancer and smoking-related pancreatic cancer [35-37], whereas for epithelial ovarian cancer there was no effect or a reduced risk[38,39].

## Conclusion

Our data indicate that variants of the BLM-TOP3A-RMI1 recombination complex, has an impact on cancer risk. Since the SNPs are common among our subjects with AML/MDS, malignant melanoma, bladder and breast cancer, even the moderate increase in risk observed in this study may be associated with a considerable impact. However, the results need to be confirmed in subsequent studies.

## Abbreviations

CI: confidence interval; FPRP: false-positive report probability; HWE: Hardy Weinberg equilibrium; Kbp: kilobasepairs; OR: odds ratio; SNP: single nucleotide polymorphism.

## Competing interests

KB, HO and MH are currently applying for a patent relating to the content of the manuscript.

## Authors' contributions

EH performed the genotyping; KE carried out the analysis on bioinformatics; EH and JB performed the statistical analysis; CI, MA, and HO have designed the case-control studies for the different cancer forms and collected the samples; KB drafted the manuscript and together with MH and HO conceived the study, and participated in its design and coordination. All authors contributed to the writing and have approved the manuscript.

## Pre-publication history

The pre-publication history for this paper can be accessed here:

http://www.biomedcentral.com/1471-2407/9/140/prepub

## Supplementary Material

Additional file 1 **Supplementary tables I–III**. Table I. Influence of all polymorphisms studied in RMI1, TOP3A, and BLM on risk for AML/MDS (acute myeloid leukemia/myelodysplastic syndromes). Table II. Influence of all polymorphisms studied RMI1, TOP3A, and BLM on risk for malignant melanoma. Table III. Influence of all polymorphisms studied in RMI1, TOP3A, and BLM on risk for bladder cancer. Click here for file
