# Supplementary material for: Association between polymorphisms in RMI1, TOP3A, and BLM and risk of cancer, a case-control study
Source: BMC Cancer. 2009 May 11;9:140. doi: 10.1186/1471-2407-9-140 (PMC2685436; doi:10.1186/1471-2407-9-140)
Supplement: Additional file 1 — Supplementary tables I–III. Table I. Influence of all polymorphisms studied in RMI1, TOP3A, and BLM on risk for AML/MDS (acute myeloid leukemia/myelodysplastic syndromes). Table II. Influence of all polymorphisms studied RMI1, TOP3A, and BLM on risk for malignant melanoma. Table III. Influence of all polymorphisms studied in RMI1, TOP3A, and BLM on risk for bladder cancer. [file 1471-2407-9-140-S1.doc]

**Supplementary tables I-III:**

Table I. Influence of all polymorphisms studied in RMI1, TOP3A, and BLM on risk for AML/MDS (acute myeloid leukemia/myelodysplastic syndromes).^a^

| Gene | SNPs  (Allelic % variant allele cases/controls) | Genotype | Cases | Controls | OR | 95% CI |
| --- | --- | --- | --- | --- | --- | --- |
| RMI1 | rs296887 ^b^  (32/25) | GG | 68 | 70 | 1.0 ^c^ | - |
|  |  | AG | 63 | 38 | **1.7** | **1.0-2.9** |
|  |  | AA | 15 | 10 | 1.5 | 0.65-3.7 |
|  | rs296891  (47/47) | CC | 40 | 32 | 1.0 | - |
|  |  | CT | 78 | 62 | 1.0 | 0.57-1.8 |
|  |  | TT | 31 | 24 | 1.0 | 0.51-2.1 |
|  | rs754144  (33/31) | GG | 68 | 55 | 1.0 | - |
|  |  | AG | 64 | 52 | 1.0 | 0.60-1.7 |
|  |  | AA | 17 | 11 | 1.3 | 0.54-2.9 |
|  | rs3737134  (26/34) | AA | 87 | 59 | 1.0 | - |
|  |  | AC | 51 | 50 | 0.7 | 0.42-1.2 |
|  |  | CC | 6 | 8 | 0.5 | 0.17-1.5 |
|  | rs6559754  (45/45) | CC | 42 | 35 | 1.0 | - |
|  |  | CG | 79 | 59 | 1.1 | 0.64-2.0 |
|  |  | GG | 28 | 24 | 0.97 | 0.48-2.0 |
|  | rs12001293  (15/16) | TT | 108 | 85 | 1.0 | - |
|  |  | AT | 37 | 31 | 0.94 | 0.54-1.6 |
|  |  | AA | 3 | 3 | 0.79 | 0.16-4.0 |
| TOP3A | rs1563634^d^  (26/34) | GG | 84 | 52 | 1.0 | - |
|  |  | AG | 54 | 52 | 0.64 | 0.38-1.1 |
|  |  | AA | 11 | 14 | 0.49 | 0.21-1.2 |
|  | rs2294913  (27/26) | GG | 74 | 67 | 1.0 | - |
|  |  | AG | 61 | 41 | 1.3 | 0.80-2.3 |
|  |  | AA | 9 | 10 | 0.82 | 0.31-2.1 |
|  | rs2294914  (10/14) | GG | 121 | 88 | 1.0 | - |
|  |  | AG | 24 | 28 | 0.62 | 0.34-1.1 |
|  |  | AA | 2 | 2 | 0.73 | 0.10-5.3 |
|  | rs4925159  (46/39) | GG | 46 | 45 | 1.0 | - |
|  |  | AG | 64 | 55 | 1.1 | 0.66-2.0 |
|  |  | AA | 35 | 18 | 1.9 | 0.94-3.8 |
|  | rs12945597  (37/25) | GG | 61 | 64 | 1.0 | - |
|  |  | AG | 63 | 49 | 1.3 | 0.81-2.3 |
|  |  | AA | 22 | 5 | **4.6** | **1.6-13** |
| BLM | rs387833  (22/22) | CC | 88 | 73 | 1.0 | - |
|  |  | CT | 54 | 39 | 1.1 | 0.69-1.9 |
|  |  | TT | 5 | 7 | 0.59 | 0.18-1.9 |
|  | rs389480  (39/42) | CC | 54 | 39 | 1.0 | - |
|  |  | CT | 73 | 60 | 0.88 | 0.52-1.5 |
|  |  | TT | 21 | 20 | 0.76 | 0.36-1.6 |
|  | rs393974  (43/39) | TT | 38 | 45 | 1.0 | - |
|  |  | GT | 82 | 44 | **2.2** | **1.3-3.9** |
|  |  | GG | 19 | 20 | 1.13 | 0.53-2.4 |
|  | rs401549  (31/30) | AA | 65 | 57 | 1.0 | - |
|  |  | AG | 75 | 51 | 1.29 | 0.78-2.1 |
|  |  | GG | 9 | 10 | 0.79 | 0.30-2.1 |
|  | rs2270132  (36/38) | TT | 58 | 45 | 1.0 | - |
|  |  | GT | 76 | 57 | 1.0 | 0.62-1.7 |
|  |  | GG | 15 | 16 | 0.73 | 0.33-1.6 |
|  | rs2518967  (25/22) | AA | 84 | 72 | 1.0 | - |
|  |  | AG | 57 | 39 | 1.3 | 0.75-2.1 |
|  |  | GG | 8 | 7 | 0.98 | 0.34-2.8 |
|  | rs2518968  (45/41) | CC | 43 | 42 | 1.0 | - |
|  |  | CG | 79 | 55 | 1.40 | 0.81-2.4 |
|  |  | GG | 27 | 21 | 1.26 | 0.62-2.6 |
|  | rs2532105  (16/12) | CC | 103 | 90 | 1.0 | - |
|  |  | CT | 43 | 25 | 1.50 | 0.85-2.7 |
|  |  | TT | 3 | 2 | 1.31 | 0.21-8.0 |
|  | rs6496724  (23/30) | AA | 86 | 58 | 1.0 | - |
|  |  | AC | 55 | 47 | 0.79 | 0.47-1.3 |
|  |  | CC | 6 | 12 | **0.34** | **0.12-0.95** |
|  | rs7165790  (38/43) | AA | 59 | 40 | 1.0 | - |
|  |  | AG | 65 | 55 | 0.80 | 0.47-1.4 |
|  |  | GG | 24 | 23 | 0.71 | 0.35-1.4 |
|  | rs7184015  (30/31) | GG | 73 | 61 | 1.0 | - |
|  |  | GT | 64 | 40 | 1.34 | 0.79-2.3 |
|  |  | TT | 12 | 17 | 0.59 | 0.26-1.3 |
|  | rs8031341  (19/24) | AA | 97 | 67 | 1.0 | - |
|  |  | AG | 48 | 45 | 0.74 | 0.44-1.2 |
|  |  | GG | 4 | 6 | 0.46 | 0.13-1.7 |
|  | rs8037430  (35/.39) | CC | 65 | 46 | 1.0 | - |
|  |  | CT | 65 | 53 | 0.87 | 0.51-1.5 |
|  |  | TT | 19 | 19 | 0.71 | 0.34-1.5 |
|  | rs16944863  (11/11) | AA | 117 | 92 | 1.0 | - |
|  |  | AG | 30 | 26 | 0.91 | 0.50-1.6 |
|  |  | GG | 1 | 0 | - | - |
|  | rs16944894  (20/22) | AA | 95 | 69 | 1.0 | - |
|  |  | AG | 49 | 45 | 0.79 | 0.48-1.3 |
|  |  | GG | 5 | 4 | 0.91 | 0.24-3.5 |

^a^ Logistic regression, unadjusted. Statistically significant associations (p ≤ 0.05) are denoted in bold.

^b^ Accession number for polymorphisms in the SNP database of National Centre of Biotechnology Information, http://www.ncbi.nlm.nih.gov/sites/entrez?db=snp

^c^ Reference category.

^d^ SNPs labelled with gray were chosen for further analysis in the breast cancer/control material. Since, the variant allele of rs1563634 was protective in this analysis, we used the variant homozygotes as reference genotype in subsequent analyses of breast cancer. The selected polymorphisms were rs1563634 (C___3063157_10, assay number for Taqman® SNP Genotyping Assays from Applied Biosystems, Foster City, CA) and rs12945597 (C__31923586_10) in *TOP3A*, rs401549 (C___2537534_1_) and rs2532105 (C___2537538_10) in *BLM*. Assay C___3063157_10 corresponds to rs7225932 which is tightly linked to rs1563634 (R^2^=0.96)

Table II. Influence of all polymorphisms studied RMI1, TOP3A, and BLM on risk for malignant melanoma.^a^

| Gene | SNPs  (Allelic % variant allele cases/controls) | Genotype | Cases | Controls | OR | 95% CI |
| --- | --- | --- | --- | --- | --- | --- |
| RMI1 | rs296887^b^  (32/25) | GG | 78 | 70 | 1.0 ^c^ | - |
|  |  | AG | 71 | 38 | **1.7** | **1.0-2.8** |
|  |  | AA | 19 | 10 | 1.7 | 0.74-3.9 |
|  | rs296891  (44/47) | CC | 54 | 32 | 1.0 | - |
|  |  | CT | 78 | 62 | 0.75 | 0.43-1.3 |
|  |  | TT | 35 | 24 | 0.86 | 0.44-1.7 |
|  | rs754144  (26/31) | GG | 95 | 55 | 1.0 | - |
|  |  | AG | 58 | 52 | 0.65 | 0.39-1.1 |
|  |  | AA | 15 | 11 | 0.79 | 0.34-1.8 |
|  | rs3737134  (22/28) | AA | 101 | 59 | 1.0 | - |
|  |  | AC | 55 | 50 | 0.64 | 0.39-1.1 |
|  |  | CC | 8 | 8 | 0.58 | 0.21-1.6 |
|  | rs6559754  (43/45) | CC | 57 | 35 | 1.0 | - |
|  |  | CG | 79 | 59 | 0.82 | 0.48-1.4 |
|  |  | GG | 32 | 24 | 0.82 | 0.42-1.6 |
|  | rs12001293  (18/16) | TT | 110 | 85 | 1.0 | - |
|  |  | AT | 55 | 31 | 1.4 | 0.81-2.3 |
|  |  | AA | 2 | 3 | 0.52 | 0.084-3.2 |
| TOP3A | rs1563634 ^d^  (25/34) | GG | 96 | 52 | 1.0 | - |
|  |  | AG | 60 | 52 | 0.63 | 0.38-1.0 |
|  |  | AA | 12 | 14 | 0.46 | 0.20-1.1 |
|  | rs2294913  (32/26) | GG | 80 | 67 | 1.0 | - |
|  |  | AG | 67 | 41 | 1.4 | 0.83-2.3 |
|  |  | AA | 20 | 10 | 1.7 | 0.73-3.8 |
|  | rs2294914  (11/14) | GG | 137 | 88 | 1.0 | - |
|  |  | AG | 24 | 28 | 0.55 | 0.30-1.0 |
|  |  | AA | 6 | 2 | 1.9 | 0.38-9.8 |
|  | rs4925159  (42/39) | GG | 59 | 45 | 1.0 | - |
|  |  | AG | 76 | 55 | 1.1 | 0.63-1.8 |
|  |  | AA | 33 | 18 | 1.4 | 0.70-2.8 |
|  | rs12945597  (32/25) | GG | 78 | 64 | 1.0 | - |
|  |  | AG | 72 | 49 | 1.2 | 0.74-2.0 |
|  |  | AA | 18 | 5 | **3.0** | **1.0-8.4** |
| BLM | rs387833  (26/22) | CC | 88 | 73 | 1.0 | - |
|  |  | CT | 71 | 39 | 1.5 | 0.92-2.5 |
|  |  | TT | 7 | 7 | 0.83 | 0.28-2.5 |
|  | rs389480  (44/42) | CC | 52 | 39 | 1.0 | - |
|  |  | CT | 82 | 60 | 1.0 | 0.60-1.7 |
|  |  | TT | 32 | 20 | 1.2 | 0.60-2.4 |
|  | rs393974  (37/39) | TT | 71 | 45 | 1.0 | - |
|  |  | GT | 64 | 44 | 0.92 | 0.54-1.6 |
|  |  | GG | 28 | 20 | 0.89 | 0.45-1.8 |
|  | rs401549  (36/30) | AA | 62 | 57 | 1.0 | - |
|  |  | AG | 90 | 51 | 1.6 | 0.99-2.7 |
|  |  | GG | 16 | 10 | 1.5 | 0.62-3.5 |
|  | rs2270132  (42/38) | TT | 56 | 45 | 1.0 | - |
|  |  | GT | 84 | 57 | 1.2 | 0.71-2.0 |
|  |  | GG | 28 | 16 | 1.4 | 0.68-3.0 |
|  | rs2518967  (26/22) | AA | 87 | 72 | 1.0 | - |
|  |  | AG | 73 | 39 | 1.5 | 0.94-2.6 |
|  |  | GG | 8 | 7 | 0.95 | 0.33-2.7 |
|  | rs2518968  (47/41) | CC | 46 | 42 | 1.0 | - |
|  |  | CG | 87 | 55 | 1.4 | 0.84-2.5 |
|  |  | GG | 35 | 21 | 1.5 | 0.77-3.0 |
|  | rs2532105  (18/12) | CC | 109 | 90 | 1.0 | - |
|  |  | CT | 56 | 25 | **1.9** | **1.1-3.2** |
|  |  | TT | 3 | 2 | 1.2 | 0.20-7.6 |
|  | rs6496724  (25/30) | AA | 94 | 58 | 1.0 | - |
|  |  | AC | 61 | 47 | 0.80 | 0.49-1.3 |
|  |  | CC | 11 | 12 | 0.57 | 0.23-1.4 |
|  | rs7165790  (38/43) | AA | 58 | 40 | 1.0 | - |
|  |  | AG | 90 | 55 | 1.1 | 0.67-1.9 |
|  |  | GG | 19 | 23 | 0.57 | 0.28-1.2 |
|  | rs7184015  (28/31) | GG | 85 | 61 | 1.0 | - |
|  |  | GT | 72 | 40 | 1.3 | 0.78-2.1 |
|  |  | TT | 11 | 17 | 0.46 | 0.20-1.1 |
|  | rs8031341  (19/24) | AA | 112 | 67 | 1.0 | - |
|  |  | AG | 48 | 45 | 0.64 | 0.38-1.1 |
|  |  | GG | 7 | 6 | 0.70 | 0.23-2.2 |
|  | rs8037430  (35/39) | CC | 65 | 46 | 1.0 | - |
|  |  | CT | 89 | 53 | 1.2 | 0.72-2.0 |
|  |  | TT | 14 | 19 | 0.52 | 0.24-1.1 |
|  | rs16944863  (13/11) | AA | 126 | 92 | 1.0 | - |
|  |  | AG | 38 | 26 | 1.1 | 0.61-1.9 |
|  |  | GG | 3 | 0 | - | - |
|  | rs16944894  (20/22) | AA | 109 | 69 | 1.0 | - |
|  |  | AG | 50 | 45 | 0.70 | 0.43-1.2 |
|  |  | GG | 9 | 4 | 1.4 | 0.42-4.8 |

^a^ Logistic regression, unadjusted. Statistically significant associations (p ≤ 0.05) are denoted in bold.

^b^ Accession number for polymorphisms in the SNP database of National Centre of Biotechnology Information, http://www.ncbi.nlm.nih.gov/sites/entrez?db=snp

^c^ Reference category.

^d^ SNPs labelled with gray were chosen for further analysis in the breast cancer/control material. Since, the variant allele of rs1563634 was protective in this analysis; we used the variant homozygotes as reference genotype in subsequent analyses of breast cancer. The selected polymorphisms were rs1563634 (C___3063157_10, assay number for Taqman® SNP Genotyping Assays from Applied Biosystems, Foster City, CA) and rs12945597 (C__31923586_10) in *TOP3A*, rs401549 (C___2537534_1_) and rs2532105 (C___2537538_10) in *BLM*. Assay C___3063157_10 corresponds to rs7225932 which is tightly linked to rs1563634 (R^2^=0.96)

Table III. Influence of all polymorphisms studied in RMI1, TOP3A, and BLM on risk for bladder cancer.^a^

| Gene | SNPs  (Allelic % variant allele cases/controls) | Genotype | Cases | Controls | OR | 95% CI |
| --- | --- | --- | --- | --- | --- | --- |
| RMI1 | rs296887^b^  (26/30) | GG | 32 | 63 | 1.0 ^c^ | - |
|  |  | AG | 23 | 68 | 0.67 | 0.35-1.3 |
|  |  | AA | 4 | 8 | 0.98 | 0.28-3.5 |
|  | rs296891  (51/43) | CC | 15 | 47 | 1.0 | - |
|  |  | CT | 29 | 65 | 1.4 | 0.68-2.9 |
|  |  | TT | 16 | 28 | 1.8 | 0.77-4.2 |
|  | rs754144  (35/30) | GG | 23 | 67 | 1.0 | - |
|  |  | AG | 32 | 61 | 1.5 | 0.81-2.9 |
|  |  | AA | 5 | 12 | 1.2 | 0.39-3.8 |
|  | rs3737134  (23/26) | AA | 35 | 76 | 1.0 | - |
|  |  | AC | 22 | 47 | 1.0 | 0.53-1.9 |
|  |  | CC | 3 | 12 | 0.54 | 0.14-2.0 |
|  | rs6559754  (50/41) | CC | 15 | 50 | 1.0 | - |
|  |  | CG | 30 | 65 | 1.5 | 0.75-3.2 |
|  |  | GG | 15 | 25 | 2.0 | 0.85-4.7 |
|  | rs12001293  (16/14) | TT | 45 | 105 | 1.0 | - |
|  |  | AT | 13 | 31 | 0.98 | 0.47-2.0 |
|  |  | AA | 3 | 4 | 1.8 | 0.38-8.1 |
| TOP3A | rs1563634 ^d^  (32/33) | GG | 25 | 66 | 1.0 | - |
|  |  | AG | 32 | 54 | 1.6 | 0.83-3.0 |
|  |  | AA | 3 | 19 | 0.42 | 0.11-1.5 |
|  | rs2294913  (25/29) | GG | 35 | 72 | 1.0 | - |
|  |  | AG | 21 | 54 | 0.80 | 0.42-1.5 |
|  |  | AA | 5 | 13 | 0.79 | 0.26-2.4 |
|  | rs2294914  (6/9) | GG | 54 | 115 | 1.0 | - |
|  |  | AG | 7 | 24 | 0.62 | 0.25-1.5 |
|  |  | AA | 0 | 0 | - | - |
|  | rs4925159  (42/38) | GG | 22 | 55 | 1.0 | - |
|  |  | AG | 24 | 62 | 0.97 | 0.49-1.9 |
|  |  | AA | 13 | 22 | 1.5 | 0.63-3.4 |
|  | rs12945597  (36/30) | GG | 26 | 73 | 1.0 | - |
|  |  | AG | 25 | 49 | 1.4 | 0.74-2.8 |
|  |  | AA | 9 | 17 | 1.5 | 0.59-3.7 |
| BLM | rs387833  (23/20) | CC | 35 | 86 | 1.0 | - |
|  |  | CT | 19 | 50 | 0.93 | 0.48-1.8 |
|  |  | TT | 4 | 2 | 4.9 | 0.86-28 |
|  | rs389480  (43/44) | CC | 18 | 41 | 1.0 | - |
|  |  | CT | 33 | 73 | 1.0 | 0.52-2.1 |
|  |  | TT | 10 | 24 | 0.95 | 0.38-2.4 |
|  | rs393974  (44/39) | TT | 20 | 53 | 1.0 | - |
|  |  | GT | 23 | 57 | 1.1 | 0.53-2.2 |
|  |  | GG | 13 | 23 | 1.5 | 0.64-3.5 |
|  | rs401549  (37/30) | AA | 26 | 64 | 1.0 | - |
|  |  | AG | 24 | 67 | 0.88 | 0.46-1.7 |
|  |  | GG | 10 | 8 | **3.1** | **1.1-8.7** |
|  | rs2270132  (40/41) | TT | 23 | 48 | 1.0 | - |
|  |  | GT | 26 | 69 | 0.79 | 0.40-1.5 |
|  |  | GG | 11 | 23 | 1.0 | 0.42-2.4 |
|  | rs2518967  (26/22) | AA | 32 | 84 | 1.0 | - |
|  |  | AG | 25 | 50 | 1.3 | 0.70-2.5 |
|  |  | GG | 3 | 6 | 1.3 | 0.31-5.6 |
|  | rs2518968  (46/43) | CC | 18 | 40 | 1.0 | - |
|  |  | CG | 29 | 79 | 0.82 | 0.41-1.6 |
|  |  | GG | 13 | 21 | 1.4 | 0.57-3.3 |
|  | rs2532105  (20/10) | CC | 40 | 111 | 1.0 | - |
|  |  | CT | 16 | 27 | 1.6 | 0.80-3.4 |
|  |  | TT | 4 | 1 | **11** | **1.2-102** |
|  | rs6496724  (23/30) | AA | 37 | 68 | 1.0 | - |
|  |  | AC | 19 | 58 | 0.60 | 0.31-1.2 |
|  |  | CC | 4 | 13 | 0.57 | 0.17-1.9 |
|  | rs7165790  (34/40) | AA | 26 | 48 | 1.0 | - |
|  |  | AG | 27 | 73 | 0.68 | 0.36-1.3 |
|  |  | GG | 7 | 19 | 0.68 | 0.25-1.8 |
|  | rs7184015  (26/25) | GG | 33 | 76 | 1.0 | - |
|  |  | GT | 21 | 56 | 0.86 | 0.45-1.6 |
|  |  | TT | 5 | 7 | 1.6 | 0.49-5.6 |
|  | rs8031341  (18/22) | AA | 41 | 85 | 1.0 | - |
|  |  | AG | 16 | 48 | 0.69 | 0.35-1.4 |
|  |  | GG | 3 | 6 | 1.0 | 0.25-4.4 |
|  | rs8037430  (28/36) | CC | 30 | 53 | 1.0 | - |
|  |  | CT | 26 | 73 | 0.63 | 0.33-1.2 |
|  |  | TT | 4 | 14 | 0.50 | 0.15-1.7 |
|  | rs16944863  (11/13) | AA | 47 | 106 | 1.0 | - |
|  |  | AG | 13 | 31 | 0.95 | 0.45-2.0 |
|  |  | GG | 0 | 2 | - | - |
|  | rs16944894  (18/20) | AA | 40 | 89 | 1.0 | - |
|  |  | AG | 18 | 45 | 0.89 | 0.46-1.7 |
|  |  | GG | 2 | 6 | 0.74 | 0.14-3.8 |

^a^ Logistic regression, unadjusted. Statistically significant associations (p ≤ 0.05) are denoted in bold.

^b^Accession number for polymorphisms in the SNP database of National Centre of Biotechnology Information, http://www.ncbi.nlm.nih.gov/sites/entrez?db=snp

^c^ Reference category.

^d^ SNPs labelled with gray were chosen for further analysis in the breast cancer/control material. The selected polymorphisms were rs1563634 (C___3063157_10, assay number for Taqman® SNP Genotyping Assays from Applied Biosystems, Foster City, CA) and rs12945597 (C__31923586_10) in *TOP3A*, rs401549 (C___2537534_1_) and rs2532105 (C___2537538_10) in *BLM*. Assay C___3063157_10 corresponds to rs7225932 which is tightly linked to rs1563634 (R^2^=0.96)
